# Specific antibody response of mice after immunization with COS-7 cell derived avian influenza virus (H5N1) recombinant proteins

**DOI:** 10.1186/1476-8518-5-10

**Published:** 2007-10-03

**Authors:** Navin Horthongkham, Tananun Srihtrakul, Niracha Athipanyasilp, Sontana Siritantikorn, Wannee Kantakamalakul, Yong Poovorawan, Ruengpung Sutthent

**Affiliations:** 1 Department of Microbiology, Faculty of Medicine Siriraj Hospital, Mahidol University, Bangkok, Thailand; 2 Department of Pediatric, Faculty of Medicine, Chulalongkorn University, Bangkok, Thailand

## Abstract

To develop avian influenza H5N1 recombinant protein, the hemagglutinin (HA), neuraminidase (NA), matrix (M), and non-structural (NS1) of avian influenza H5N1 isolates from Thailand were engineered to be expressed in prokaryotic (*E. coli*) and mammalian cell (COS-7) system. The plasmid pBAD-His and pSec-His were used as vectors for these inserted genes. Mice immunized with purified recombinant proteins at concentration 50–250 μg intramuscularly with Alum adjuvant at week 0, week 2, and week 3 showed a good immunogenicity measured by ELISA and neutralization assay. The HA and NS recombinant proteins produced in COS-7 cells can induce specific antibody titer detected by neutralization assay significantly higher than corresponding recombinant proteins produced in *E. coli *system. The antibody produced in immunized mice could neutralize heterologous avian influenza virus determined by micro-neutralization assay. This study shows that avian influenza virus H5N1 recombinant proteins produced in mammalian cell system were able to induce neutralizing antibody response.

## Introduction

From January 2004, the pandemic of highly pathogenic avian influenza H5N1 (AI) in poultry and human had started from 9 Asian countries, such as Cambodia, China, Indonesia, Japan, Laos, Malaysia, South Korea, Thailand, and Vietnam [1]. It has expanded worldwide. In Thailand, a total of 22 human infected cases were reported until present with the last case detected in November 2005. The development of prevention avian influenza vaccine was ongoing by based on concept of influenza vaccine including inactivated or subunit virus grown in embryonated chicken eggs and recombinant technology including DNA, peptide, recombinant protein, live vector vaccines [2-6]. However, concerns about safety, mass production, preexisting immunity in people, immune responses against vector itself, the use of purified recombinant avian influenza hemagglutinin and neuraminidase proteins appear to be a promising alternative. The H5N1 vaccines were developed and trial. The controversial of using avian influenza vaccine in the poultry is still under discussion in Thailand.

Because hemagglutinin (HA) protein is a major viral surface antigen against neutralizing antibodies elicited, recombinant HA was a target as a candidate avian influenza vaccine. The mammalian cell (COS-7 cell line) and prokaryotic cell (E. coli) were used as the expression cell system for recombinant HA protein production. Also, the recombinant neuraminidase (NA) protein, the other viral surface protein, and nucleocapsid protein (M), and non-structural (NS1) protein, were also produced. The purified proteins, rHA5, rNA1, rNS1, and rM, produced from *E. coli *and COS-7 cellls, were administered in mice in combination with adjuvant, was capable of eliciting antibody specific for avian influenza virus, detected by ELISA and neutralizing antibody assay.

## Materials and methods

### Virus

Avian influenza virus (H5N1) isolates from Thailand were selected and the nucleotide sequences of hemagglutinin (HA), neuraminidase (NA), matrix (M), and non-structural (NS) genes were identified as H5 and N1 with the accession number: A/Thailand/HA20/2005 (DQ885618), A/Thailand/M38/2005 (DQ885619Q), A/Thailand/NA60/2005 (DQ885620), and A/Thailand/NS49/2005 (DQ885621), respectively. All viruses were grown in MDCK cell line and processed in biosafety level 3 containment by trained lab technicians. Viral RNA was extracted from culture supernatant by using QiaAamp viral RNA mini kit (Qiagen, Germany).

### Cloning of avian influenza virus genes (HA, NA, NS, M)

After cDNA was amplified from viral RNA lysate with universal primer (5'-AGCAAAAGCAGG-3') by RT-PCR using Superscript III One step RT PCR (Invitrogen, USA). PCR was used to amplify HA gene with forward primer (5'-CTC GAG GAT ATC CAA AAG CAG GGG TCC GAT CT-3') and reverse primer (5'-AAG CTT GCG GCC GCC AAT GAC CCA TTG GAA CA-3'), NA gene with forward primer (5'-CTG CAG AAG CTT AGC AAA AGC AGG AGT-3') and reverse primer (5'-GAA TTC GCG GCC GCG TAC TTG TCA ATG GTG A-3'), M gene with forward primer (5'-GAG CTC GAT ATC ATG AGT CTT CTA ACC GAG GTC-3') and reverse primer (5'-GAA TTC GCG GCC GCC TTG AAT CGC TGC ATT TGC AC-3'), and NS gene with forward primer (5'-CTC GAG GAT ATC AGC AAA AGC AGG GTG-3') and reverse primer (5'-GAA TTC GCG GCC GCC CAT CTT ATC TCT TGA-3'). The expected amplified size of HA, NA, M, and NS1 genes are 1778 bps, 1413 bps, 1027 bps, and 890 bps, respectively.

PCR was performed for 3 cycles, each consisted of 94°C denaturation step for 1 min (6 min for first cycle), 55°C annealing step for 1 min, and 72°C extension step for 1 min, followed by 31 cycles of 94°C for 15 sec, 55°C for 45 sec, 72°C for 90 sec and the final extension at 72°C for 10 min in both of first round and second round PCR. The amplified products were cloned into vector pGEM-T (Promega, USA) and subcloned into pBAD/His C vector (Invitrogen, USA.) and used to transform LMG194 competent *E. coli *cells. All colonies of *E. coli *containing pBAD/His-HA, pBAD/His-NA, pBAD/His-M, pBAD/His-NS1, were checked for positive clones containing insert fragment of and by digestion plasmid DNA with restriction enzymes, *Pst *I and *Eco*RI.

To construct the mammalian expression vector, pSecTag2/Hygro C (Invitrogen, USA.) for HA, NA, M, and NS1 protein expression in COS-7 cell line, the XhoI/ApaI digested DNA from pBAD/His-HA or pBAD/His-NA or pBAD/His-NS or pBAD/His-M was subcloned into digested pSecTag2/Hygro C vector to produce pSec-His-HA, pSec-His-NS1 and pSec-His-NA. The pBAD/His-HA, pBAD/His-NA, pBAD/His-NS1, pBAD/His-M DNA was used for transformation into DH5α competent *E. Coli *cells and pSec-His-HA, pSec-His-NS1 and pSec-His-NA DNA to transform COS-7 cell line by using polyfect transfection system (Qiagen, USA).

### Recombinant protein expression and purification [7,8]

Overnight culture of *E. coli *strain LMG containing pBAD/His-HA or pBAD/His-NA or pBAD/His-NS1 or pBAD/His-M was added to a final of 0.2% to induce the production of polyhistidine tagged protein and the recombinant protein was extracted and purified by metal affinity column, MagneHis™ protein purification system (Promega, USA), to purify the polyhistidine tagged protein.

The stably expressed pSec-His-HA, pSec-His-NS1, pSec-His-M and pSec-His-NA in COS-7 cell lines in medium containing 200 μg/ml of hygromycin B were lysed with lysozyme. The cell lysate was used to purify recombinant protein with MagneHis™ protein purification system (Promega, USA). The recombinant HA, NA, NS, M proteins were detected by SDS-PAGE analysis (3.85% stacking gel and 10% separating gel with a constant voltage of 150 volts for 1 hour) and followed by Western blot analysis against mouse anti-Xpress serum (Invitrogen, USA) as previously described [8].

### Immunization of mice

Animal usage in this study was performed according to the national guidelines and instructional policies. Mice were purchased from the National Laboratory Animal Center, Thailand. Six to eight-week-old pathogen-free, female Balb/c mice were used for vaccination. The animals were housed in a temperature controlled environment at 22–24°C with 12 h day-night cycles, and received food and water *ad libitum*. Mice were immunized three times intramuscularly (IM) at 1-week interval with 200 μl doses of 50, 100, 150, 200, and 250 μg of rHA, rNA, rNS1, and rM protein produced in *E. coli *and COS-7 cells plus an emulsion prepared with Alum adjuvant. Two mice were injected with pSecHis/HygroC vector protein as control. Boosts were given at 2 and 3 weeks after the first immunization. One week after the last boosting, mice were sacrificed and whole blood was collected for immunogenicity analysis. Then, 250 μg of rHA, rNA, rNS1, and rM protein produced in *E. coli *and COS-7 cells were selected to immunize 5 groups of mice (5 mice/group) for each protein and boosted as described. Serum was prepared by centrifugation of clotted blood at 1800 × g for 5 min, stored at -80°C until used.

### Detection of H5N1 specific antibody from immunized mice sera [9]

#### ELISA

The presence of serum anti-HA, -NA, -NS1, -M specific immunoglobulins was determined by an enzyme linked immunosorbent assay (ELISA). Briefly, 500 μl of purified HA or NA, or NS or M proteins were incubated with 50 μl of MagnaHis bead (Promega, USA) and 5 μg/ml of bead solution was added to each 96-well plates. Diluted mice sera in blocking solution (PBS/Tween 20 containing 5% skim milk) were added after 5 times washing. The horseradish peroxidase-labeled goat anti-mouse Ig(G+M+A) diluted 1:1000 in blocking solution and 100 μl of TMB substrate were used for ELISA. Reactions were stopped by adding 100 μl of 1 N H_2_SO_4_. The absorbance was measured at 450 nm with an ELISA microplate reader. The cut off value of absorbance was calculated as formula: cut off = 0.124 [(X+3SD) × 2], X = mean of all negative samples absorbance +3 standard deviation) × 2. ELISA index (EI = Absorbance/cut off) is a ratio of absorbance value of any sera and cut off value. EI of any area is less than 1, interpretation is negative, and EI ≥ 1 means positive result.

#### Micro neutralization assay [10]

The H5N1 virus, A/Thailand/RPNP/2005 (DQ885616) with tissue infectious dosage 50 (TCID_50_) per ml were incubated with diluted mice serum samples twofold in medium, from 1:4 to 1:2560. Mixtures of virus and serum were transferred to monolayers of MDCK cells and incubated for one hour at 37°C in 5% CO_2 _for 3 days. After three days, cell medium was incubated with 100 μl of monoclonal antibodies directed against the influenza type A or type B nucleocapsid antigen (Chemicon Europe, Hampshire, UK). After incubation for 60 min at 37°C and washing, affinity-purified peroxidase-conjugated, goat anti-mouse IgG (Jackson ImmunoResearch Europe, Cambridgeshire, UK), was added and the plates were incubated at room temperature for 120 min. After being washed, 100 μl substrate (orthophenylenediamine) was added, and the enzyme reaction was stopped after 30 min with 100 μl 2.5 M sulfuric acid. The reaction was quantified by measuring the OD at wavelength 492 nm. The neutralization (NT) titers were defined as the inverted value of the serum dilution giving ≥ 50% OD reduction compared to the virus control.

## Result

### Characterization of HA, NA, NS, and M recombinant proteins from *E. coli *and Cos7 cell system

**Figure 1:**
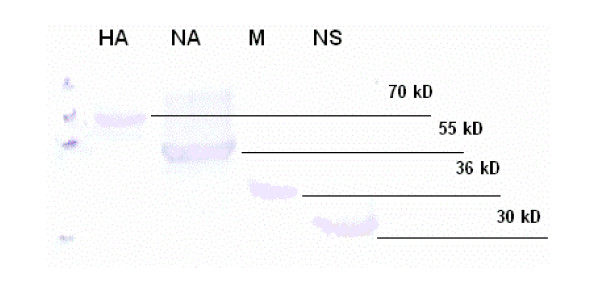
Immunoblot analysis of recombinant HA, NA, M, NS1 proteins produced in COS-7 cells against anti-Xpress antibody. The molecular weight of recombinant HA, NA, M, and NS1 were shown in kD as 70, 55, 36, and 30 KD, respectively.

The recombinant proteins were purified by affinity chromatography using paramagenetic precharged nickel particles (MagneHis™ Ni-particles). The 63, 50, 30, and 26 Kdal of recombinant HA, NA, M, and NS1 protein produced in *E. coli *system were detected by immunoblot hybridization assay against anti-Xpress antibody. Then, these HA, NA, M and NS genes in *E. coli *plasmid system were transferred to pSec/His mammalian cell system. The 70, 55, 36, and 30 Kdal of HA, NA, M, NS1 recombinant protein produced in mammalian cell system were detected by Western blot hybridization against anti-Xpress antibody as shown in Fig 1. Only the recombinant proteins from the clones that were expressed in both *E. coli *and mammalian system were used for further immunogenicity study. The yield of recombinant proteins HA (rHA5) and NA (rNA1) produced by pBAD-His-HA, pBAD-His-NA in *E. coli *system and pSec-His-HA and pSec-His-NA in mammalian cell system were 0.5 mg per 100 ml bacterial culture and 0.05 mg per 100 ml cells, respectively.

### Immunity in immunized mice

**Figure 2:**
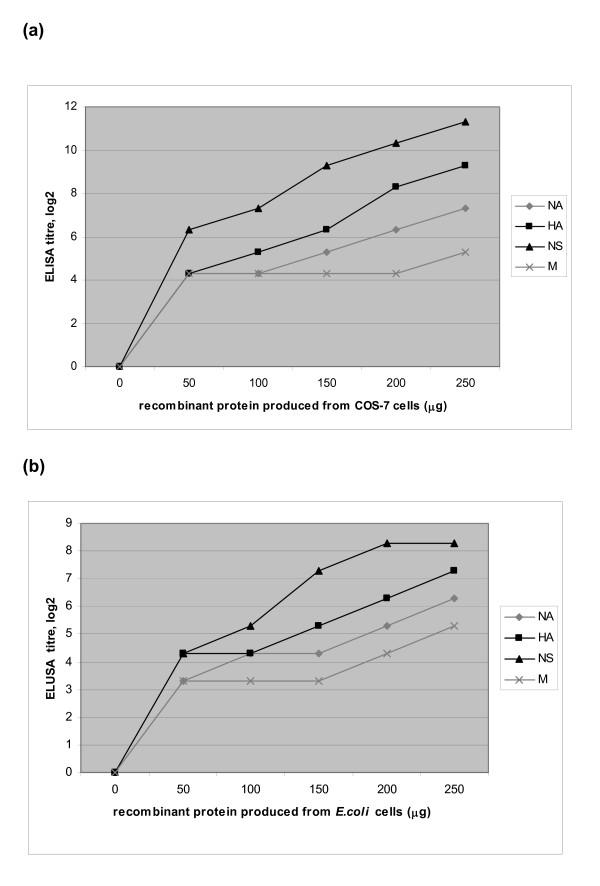
Dose responsive curve of antibody response in immunized mice. Sera from 5 mice per group were collected 1 week after last immunization and tested by ELISA for the presence of specific antibodies by using recombinant HA or NA, or NS or M proteins. Antibody titers are expressed as the log_2 _values of reciprocal endpoint titers. (a) Antibody titers of sera from mice immunized with recombinant HA, NA, M, and NS proteins produced in mammalian (COS-7) cell system; 50, 100, 150, 200 and 250 μg. (b) Antibody titers of sera from mice immunized with recombinant HA, NA, M, and NS proteins produced in prokaryotic (*E. coli*) cell system; 50, 100, 150, 200 and 250 μg.

To determine the optimal concentration of HA, NA, M, NS recombinant proteins to induce immunogenicity, the recombinant proteins at 50–250 microgram were used to immunize mice. The specific antibody response against avian influenza virus antigen of HA, NA, M, NS1 recombinant protein immunized mice was determined by ELISA and shown in dose responsive curve in Fig 2. The NS1 recombinant protein produced from COS-7 cells (rNS/COS-7) gave highest antibody response titer measured by ELISA at dose 250 μg, while M recombinant protein gave lowest immunogenicity even increasing dose to 250 μg. The optimal dose at 250 μg of all recombinant proteins with Alum adjuvant was selected to use for comparing the specific antibody elicit in mice determined by ELISA and neutralization antibody assay. The HA and NS recombinant proteins produced in COS-7 cells can induce specific antibody titer detected by neutralization assay significantly higher than corresponding recombinant proteins produced in *E. coli *system at 250 μg as shown in Table 1. The NS1/COS-7 cells recombinant protein can induce ELISA and neutralizing antibody titer higher significantly than any other recombinant proteins. Even the antibody response measured by ELISA in rNA protein/COS-7 cells immunized mice was significantly higher than that produced in *E. coli *system, but their neutralizing antibody responses were not different. This result shows that avian influenza virus H5N1 heterologous strain could be neutralized by recombinant HA, NA, and NS proteins immunized sera from mice *in vitro*.

**Table 1:** Neutralizing antibody titer (NT titer) (geometric mean) of sera from immunized mice against heterologous H5N1 influenza virus (A/Thailand/1/RPNP/2005). Sera from 5 groups of 5 mice per recombinant protein immunization were collected 1 week after last immunization with 250 μg of recombinant HA or NA, or NS1 or M proteins. Mann-Whitney method

**Recombinant proteins**	**Neutralization titer**		**ELISA titer**	
	
	GMT (± SD)	P	GMT (± SD)	P
NA/COS-7	34 (± 8)	0.36	160 (± 78)	0.01
NA/*E. coli*	13 (± 5)		60 (± 21)	
HA/COS-7	91.8 (± 35.7)	0.01	422 (± 175)	0.28
HA/*E. coli*	22 (± 8)		160 (± 87)	
NS1/COS-7	139* (± 35)	0.01	1688* (± 701)	0.01
NS1/*E. coli*	22 (± 8)		422 (± 175)	
M/COS-7	0	NA	40 (± 21)	0.1
M/*E. coli*	0		22 (± 8)	

*Significant (P < 0.05, Mann-Whitney method, NS/COS-7 and other proteins)

## Discussion

The mammalian COS-7 cell system has successfully used as host for the efficient production of avian influenza virus (H5N1) proteins including hemagglutinin (HA), neuraminidase (NA), matrix (M), and non-structural (NS1) proteins with yield of 0.05 mg per 100 ml cells. These recombinant proteins could elicit specific antibody response against avian influenza virus (H5N1) antigen tested by ELISA. The protecting antibody *in vitro*, which was determined by neutralizing antibody assay, was also developed in animal immunized with HA, NA and NS recombinant proteins. Comparing between recombinant proteins produced in *E. coli *and COS-7 cell, we found that at the same concentration, recombinant protein produced from COS-7 cells could induce significantly higher antibody response measured by ELISA and neutralization assay. The recombinant viral proteins production from mammalian cell system are modified similarly to those naturally produced in viral infected human cells [11,12]. So, the antigenic epitopes are not different from the challenging heterologous virus used in neutralization assay.

However, recombinant NS protein produced from COS-7 cells showed highest antibody response measured by ELISA and neutralization assay. The NS1 protein is encoded in the shortest segment of the viral genome and is abundant in influenza virus-infected cells, but it has not been detected in virions [13]. The protein is found predominantly in the nucleus and has pleiotropic activities such as shutting off the host protein synthesis, supporting the translation of the late viral proteins, inhibiting pre-mRNA splicing, regulating the nuclear transport of mRNA, or exhibiting interferon antagonistic activity.

We have shown that immunogenic potential of recombinant HA, NA and NS1 proteins produced from COS-7 cells as described here, may be appropriate for further development of an avian influenza virus vaccine that could elicit the cross-reactive neutralizing antibody. This result was preliminary for further proof by challenging study in animals.
